# Prader-Willi syndrome: endocrine manifestations and management

**DOI:** 10.20945/2359-3997000000248

**Published:** 2020-06-05

**Authors:** Crésio Alves, Ruth Rocha Franco

**Affiliations:** 1 Hospital Universitário Prof. Edgard Santos Faculdade de Medicina Universidade Federal da Bahia Salvador BA Brasil Unidade de Endocrinologia Pediátrica, Hospital Universitário Prof. Edgard Santos, Faculdade de Medicina, Universidade Federal da Bahia (UFBA), Salvador, BA, Brasil; 2 Hospital das Clínicas Faculdade de Medicina Universidade de São Paulo São Paulo SP Brasil Ambulatório de Prader-Willi, Instituto da Criança, Hospital das Clínicas, Faculdade de Medicina, Universidade de São Paulo (USP), São Paulo, SP, Brasil

**Keywords:** Prader-Willi syndrome, growth hormone, hypogonadism, hypothyroidism, adrenal insufficiency, glucose abnormalities

## Abstract

Prader-Willi syndrome (PWS) is a genetic disorder caused by the absence of gene expression in the 15q11.2-q13 paternal chromosome. Patients with PWS develop hypothalamic dysfunction that can lead to various endocrine changes such as: obesity, growth hormone deficiency, hypogonadism, hypothyroidism, adrenal insufficiency and low bone mineral density. In addition, individuals with PWS have increased risk of developing type 2 diabetes mellitus. This review summarizes and updates the current knowledge about the prevention, diagnosis and treatment of endocrine manifestations associated with Prader Willi syndrome, especially diagnosis of growth hormone deficiency, management and monitoring of adverse effects; diagnosis of central adrenal insufficiency and management in stressful situations; screening for central hypothyroidism; research and treatment of hypogonadism; prevention and treatment of disorders of glucose metabolism. Careful attention to the endocrine aspects of PWS contributes significantly to the health of these individuals. Arch Endocrinol Metab. 2020;64(3):223-34

## INTRODUCTION

Prader-Willi syndrome (PWS) is a genetic syndrome, with no predilection for race or sex, with an estimated prevalence of 1: 10,000-1: 30,000 in different populations studied ( 1 ).

It is caused by the absence of paternal gene expression on chromosomal region 15q11.2-q13c ( 1 ). In this region, there are active genes with exclusively paternal expression, while these same genes in the maternal allele are inactivated by epigenetic mechanisms and are not expressed in individuals with SPW ( 2 ). There are 3 main classes of chromosomal abnormalities that lead to PWS: deletion, maternal disomy (UPD), and impring defect (ID). Genic mutation and balanced translocation can also be found ( 2 ). The most common genetic mechanism is a 5 to 6 Mb deletion, occuring in 65%-75% of individuals with SPW^3^The second most common genetic mechanism is UPD, reported in 20%-30% of individuals and the third mechanism, is a genomic defect in the region that controls the imprinting process, occuring in 1%-3% of individuals with SPW1 ( 1 - 3 ) .

Other genes with paternal expression located at the end of the 15q11-q13 region may contribute to the PWS phenotype, such as *MKRN3, MAGEL2, NDN* and *C15orf2* ( 3 ). Several of these genes are involved in neural development, brain function, infertility, and circadian rhythm. *SNORD 116* is expressed in the hypothalamic appetite control centers ( 3 ). Deletion of *SNORD 116* l causes pro convertase 1 (PC1) deficiency ( 4 ). PCI deficiency may explain the major hormonal changes found in PWS: GhRH (growth hormone deficiency and short stature); proGnRH (hypogonadism); progrelin (hyperghelinemia); proinsulin (relative hypoinsulinemia and type 2 diabetes mellitus); proopiomelanocortin – POMC (hypocortisolism); and ProTRH (hypothyroidism) ( 4 ). Many other hormones involved in food intake and energy expenditure are processed by PC1, including agouti gene-related peptide prohormones ( *AgRP* ), neuropeptide Y ( *NPY* ), oxytocin, and brain-derived neurotrophic factor ( *BDNF* ) ( 4 ).

Therefore, individuals with PWS develop hypothalamic dysfunction that can lead to various endocrine disorders. This review aims to update the endocrinological aspects of PWS including its screening, diagnosis and treatment.

## PWS: MAIN CLINICAL MANIFESTATIONS

The clinical manifestations of PWS present great variability in clinical expression and vary according to the age of presentation:

- Prenatal period: reduced fetal activity (88%), polyhydramnios (34%) reflecting the inability to coordinate suction and swallowing, pelvic presentation, preterm delivery, small for gestational age (65%) and increased head/abdomen circumference ratio (43%) ( 5 , 6 );
- 0-2 years: hypotonia, poor sucking, poor crying, failure to thrive, difficulty feeding with need for gastric tube or gastrostomy (in some children), cryptorchidism, genital hypoplasia (scrotal hypoplasia, hypoplasia of the clitoris and large or small vaginal lips), developmental delay;
- 2-4 years: hypotonia, recovery of growth with onset of weight gain without change in dietary pattern and developmental delay;
- 4-8 years: hyperphagia, progressive obesity, developmental delay;
- > 8 years: severe hyperphagia, obsession with food (eating spoiled or junk food), obstructive sleep apnea, behavioral disorder (fury attacks, stubbornness, kleptomania, obsessive disorder, affective disorder), cognitive deficit, nearsightedness, increased pain threshold, self-injurious skin lesions, temperature instability, viscous saliva, decreased ability to vomit, gastric distension leading to stomach rupture. Clear evidence of dysmorphia (dolichocepaly, almond shaped eyes, strabimus, hin and inverted down upper lip, narrow nasal bridge, dental enamel hypoplasia, small hands and feet), scoliosis and increased risk of choking ( 1 , 7 ).

## PWS: CLINICAL MANIFESTATIONS OF THE DIFFERENT GENETIC TYPES

Several studies have described clinical differences between genetic subtypes in PWS, which are described in detail, in the following topics ( 6 - 10 ).

### Deletion subtype

The deletion subtype can be subdivided into two types according to the size of the deletion: type I and type II. In type I deletion, the loss is 6bMb, which is 500 kb larger than the type II deletion. Atypical deletions occur in 7% to 9% of cases and may be larger or smaller than type I or type II ( 2 ).

The type I deletion includes 4 more genes ( *NIPA1, NIPA2, CYFIP1, GCP5* ) ( 11 ). These 4 genes, when absent, are associated with worse neurological and cognitive development; increased food compulsion; difficult behavior; sleep disorders and impaired speech articulation ( 11 ). Therefore, patients with type I deletion have a more severe phenotype than type II.

### Maternal dissomy subtype

In this subtype, both chromosome 15 alleles are inherited from the mother, accordingly to the pathogenetic mechanism, it can be classified in three subtypes:

- Maternal heterodysomy: two different maternal alleles due to meiosis I errors and no homologous chromosomal disjunction;
- Maternal isodisomy: two identical maternal alleles due to errors in meiosis II due to non-disjunction;
- Segmental isodisomy: two partially different mother alleles due to meiosis I errors from events without disjunction and crossover of isodisomy segments or loss of heterozygosity ( 2 , 7 , 12 ).

Individuals with PWS with UPD by isodisomy or segmental isodisomy have a higher risk of having secondary genetic conditions that involve recessive disease genes on chromosome 15, if the mother carries one of these genes. Several hundred recessive genes are located on chromosome 15 with the potential of causing hearing loss, cardiac abnormalities, seizures or metabolic defects. In addition, women with UPD due to trisomic salvage in early pregnancy may develop X-linked genetic conditions ( 12 - 15 ).

Currently, the older maternal age is more prevalent in westernized societies and this can also affect the frequency of genetics in PWS ( 16 ). One of the hypothesis is a greater relationship between UPD and advanced maternal age, attributed to non-disjunction during meiosis, forming a trisomic zygote with subsequent loss of the paternal chromosome through a rescue event early in pregnancy ( 2 ).

Individuals with UPD have a higher verbal intelligence coefficient and milder behavioral problems but are more likely to have psychosis in later life ( 17 ).

### Imprinting defect subtype

Patients with imprinting defect subtype have clinical features similar to the UPD type ( 17 ).

## PWS: ENDOCRINE MANIFESTATIONS

The main endocrine abnormalities of PWS, detailed in the following paragraphs, are: growth hormone deficiency; gonadotrophins deficiency and/or gonadal disorders; obesity; metabolic syndrome; early pubarche and precocious puberty; central hypothyroidism; central adrenal insufficiency; type 2 diabetes mellitus and low bone mineral density ( Table 1 ) ( 7 , 18 )

**Table 1:** Prader-Willi syndrome: diagnosis and treatment of endocrine manifestations

Endocrine findings	Diagnosis	Treatment
Growth hormone deficiency	Height, Weight, Body mass index, Waist circumference, Growth velocity, IGF-1, IGFBP-3, Growth hormone stimulation test (if indicated), Polysomnography	Recombinant human growth hormone
Obesity and Metabolic Syndrome	Height, Weight, Body mass index, Waist circumference, Blood pressure Lipid profile, Blood glucose, Insulin, Liver enzymes, Urea, Creatinine, Uric acid, C reactive protein	Caloric and carbohydrate restriction, Physical activity
Type 2 diabetes mellitus	Oral glucose tolerance test, Fasting blood glucose, HbA1c	Diet, Metformin, Insulin, Liraglutide, Exenatide
Hypogonadism	LH, FSH, Testosterone/Estradiol, Inhibin B, Bone age	– Females: 17-Beta-estradiol (oral). When pubertal stage M4/M5, replace with oral hormonal contraceptive – Boys: Testosterone (intramuscular)
Hypothyroidism	TSH, FreeT4	Levothyroxine
Adrenal insufficiency	ACTH and Cortisol during stress situations, or after ACTH stimulation test	Hydrocortisone acetate: continuous or during stress
Premature adrenarche	DHEA, S-DHEA, Androstenedione, 17-OH progesterone	Clinical follow-up
Precocious puberty	LH, FSH, Testosterone/Estradiol	GnRH Analog
Low bone mineral density	Bone Densitometry, 25(OH) Vitamin D, Calcium, Phosphorus, Magnesium, Alkaline Phosphatase, Albumin	Physical activity, Vitamin D and Calcium

ACTH: adrenocorticotrophic hormone; FSH: follicle stimulating hormone; GH: growth hormone; HbA1c: glycated hemoglobin; IGF-1: insulin-like growth factor 1; LH: luteinizing hormone; GH: growth hormone; S-DHEA: dehydroepiandrosterone sulfate; TSH: thyrotrophic hormone; FT4: free thyroxine.

## GROWTH HORMONE DEFICIENCY

Growth hormone deficiency (GHD) is present in 40%-100% of patients ( 19 , 20 ). In the KIGS International database, it was detected that 74% of the children with PWS had DGH ( 21 ). Short stature is common and without growth hormone replacement, mean final height is 155-160 cm for men and 145-150 cm for women ( 21 ). Younger children may have a normal growth hormone pituitary reserve but are deficient in the hypothalamic secretion of GH releasing hormone. As these children grow-up they may run out of this GH reserve and become disabled. Thus, GHD is considered to be an evolutionary process in individuals with PWS ( 22 ). Stimulation tests for GH dosing are not mandatory in children and adolescents with molecular diagnosis of PWS because most of them have GH deficiency or GH/IGF-1 axis dysfunction. However, whenever possible, it is recommended to evaluate the GH-IGF axis to meet individual product dispensing requirements. This evaluation should be done by a multidisciplinary team with knowledge of PWS to diagnose and treat comorbidities that may affect the safety and response to human recombinant growth hormone (rhGH).

Few studies have compared the effectiveness of GH therapy in the different genetic types of PWS. Regarding the GH secretory pattern, patients with deletion reach higher peaks after clonidine stimulation, arginine test and insulin test than patients with UPD ( 23 , 24 ). Interestingly, patients in transition with UPD have a higher incidence of GHD than those with deletion (80% *versus* 25%) ( 25 ). Assessment of pituitary GH secretion shows a later GH response in UPD individuals ( 26 ). On the other hand, stimulated GH and GH secretion are similar in both individuals with type I and type II deletion ( 26 ). The results show that the GH group presents significantly higher verbal IQ scores than the non-GH group. This suggests that GH therapy improves intelligence in patients with PWS ( 26 ). There is no significant difference in total IQ scores in individuals with deletion compared with UPD. However, GH therapy in the UPD subtype results in less decline in IQ scores with age ( 26 ). Verbal IQ scores are significantly higher in the UPD subtype than in the deletion, with better development of verbal than spatial skills.

Treatment with rhGH for children with PWS was first described in 1987. In the year 2000, rhGH for PWS was approved in the United States by the Federal Drug Administration (FDA) and in 2001 use was approved in Europe, by the European Medicine Agency (EMA). In Brazil, each state has its own protocol for the delivery of rhGH and, patients with PWS are not always inclued in these criteria.

The beneficial effects of rhGH on PWS substantially improved the phenotype of these individuals ( 21 ); including: decreased fat mass, increased muscle mass, increased growth rate (up to 16 cm increase in final height), normalization of cranial diameter, improvement of bone mineral density, dyslipidemia, development, quality of life, and physical performance ( 20 , 21 ).

Despite the recommendation to initiate rhGH replacement in PWS at a mean age of 7 years, rhGH treatment has been indicated earlier ( 27 ). The benefits of treatment with rhGH starting at 4-6 months of age have already been demonstrated ( 28 ). Recently, studies have shown positive effects on neurocognitive development and behavior when rhGH is started before 2 years of age ( 29 , 30 ). Infants and children should initiate rhGH replacement with a daily dose of 0.5 mg/m^2^/day, with subsequent adjustments up to 1.0 mg/m^2^/day (0.035 mg/kg/day or 0.245 mg/kg/week) over a period of 3-6 months ( 31 ). The efficacy of doses of less than 1.0 mg/m^2^/day given over a long period of time is still uncertain ( 32 ).

Patients with PWS are highly sensitive to rhGH in terms of IGF-1 generation and standard doses often result in IGF-1 levels outside the normal range, especially in the first 6 months of treatment ( 33 ). Although total IGF-1 levels are elevated, free IGF-1 levels are similar, thus decreasing the risks associated with elevation of total IGF-1 ( 33 ). IGF-1 normative ranges for age and sex, are laboratory and assay dependent. An IGF-1 < 0 SDS in infants or children with a proven genetic diagnosis of PWS may indicate growth hormone deficiency ( 31 , 34 ). IGF-1 levels may be altered by nutritional status, disease, ethnicity, estrogen presence, physical activity, stress, insulin levels, and time of day. Therefore, it is also recommended to measure the IGFBP-3 level and calculate the IGF-1/IGFBP-3 molar ratio to determine IGF-1 bioavailability ( 5 ). Children with PWS have elevated IGF-1 levels after 2 years of GH treatment. The GH dose should be assessed using the calculated level of bioavailable IGF-1 instead of total IGF1 ( 5 ).

Children with PWS have a higher incidence of central apnea and obstructive apnea. As rhGH therapy may lead to lymphoid tissue growth, parents should be informed about the potential association between rhGH therapy and worsening obstructive apnea. Therefore, polysomnography should be performed prior to initiation of therapy within the first 3-6 months after initiation of rhGH therapy and annually thereafter. Patients with obstructive sleep apnea should be evaluated by otolaryngologists to assess indication of adenotonsillectomy; and if they present more than 5 central apneas/hour, they should be evaluated by a neurologist ( 35 ).

Although rhGH should not be initiated during an acute respiratory infection, it is not be necessary to discontinue use during subsequent episodes of respiratory infection. Scoliosis is a common complication in PWS, but neither incidence nor rate of progression is influenced by rhGH treatment ( 31 ).

Adults with PWS should receive an initial dose of rhGH of 0.1-1.6 mg/day. Subsequent titration should be based on IGF-I levels, clinical response, age and sex. Studies indicate that, in adults with PWS, the optimal level of IGF-I during rhGH treatment is between 0 and +2 SDS for age to achieve the beneficial effects and at the same time the lowest possible risk of adverse events ( 30 , 32 , 36 ).

The rhGH is contraindicated in the following situations: severe acute illness; severe obesity, uncontrolled diabetes mellitus, severe obstructive sleep apnea, active proliferative diseases; hypersensitivity to the product and psychosisdeal ( 31 ).

The safety of rhGH is a major concern in people with PWS. A recent study evaluated 522 pre-pubertal children treated with rhGH for three years and 173 children who reached final height. The safety analysis included 2332 children and data were collected from KIGS records from 1987 to 2012 ( 33 ). To date, no concrete association between rhGH and cancer has been confirmed ( 37 ). The findings showed that rhGH therapy is not associated with the onset of leukemia in patients without risk factors for leukemia ( 37 ). After 15 years of the use of rhGH in patients with PWS, the conclusion was that the benefit/risk ratio is positive ( 38 , 39 ). The association between rhGH treatment and changes in sleep in adults with PWS is limited. A single study reported that one in ten individuals had worsened apnea/hypopnea index after 6 weeks of therapy ( 39 ). Obese adults with PWS were identified as having hypokinetic cardiac characteristics similar to those observed in individuals with GH deficiency (i.e., lower systolic function, decreased cardiac mass and decreased chronotropic response to an adrenergic stimulus) ( 39 ).

The side effects of rhGH treatment described in PWS are: peripheral edema, joint pain, sleep apnea, disordered breathing, snoring, respiratory pauses, excessive daytime sleepiness, cerebral pseudotumor, benign intracranial hypertension, headache, visual changes, nausea, dizziness, femoral head epiphysiolysis, hip and/or knee pain, gait disturbances, and T4 level decrease ( 29 ).

Tolerability of rhGH by pediatric and adult patients with PWS is high. For children with PWS treated with rhGH and followed in phase 4 post-marketing research, the reported rate of side effects leading to cessation of treatment in general trials is low ( 31 ).

## HYPOGONADISM

The etiology of hypogonadism in PWS is quite heterogeneous and can be caused by defect in the hypothalamic-pituitary axis, failure of the gonad response or the combination of the two forms ( 40 ). Hypogonadism may be present in both sexes as genital hypoplasia, lack of pubertal development or partial pubertal development and infertility.

Girls may have hypoplasia of small lips and clitoris. Although hypogonadism is a hallmark, there are reported cases of spontaneous gestation, with a higher risk of having children with Angelman’s syndrome ( 41 , 42 ). Therefore, sexual counseling and advice on contraception should be provided ( 41 ). Systematic studies evaluating the benefits and safety of hormone replacement in female adolescents or adults with PWS are not yet available. But the recommendation is to prescribe the hormonal replacement as it is done in Turner syndrome ( 5 ). If an estradiol patch is prescribed, the initial dose is 0.1 µg/kg, applying at night, and withdrawing in the morning. After 6 months, switch to 0.1 µg/kg throughout all day. After 12 months, the dose may be increased to 0.2 µg/kg. When bleeding occurs, start oral progesterone (5 mg, PO, 12 days/month) or oral contraceptive pills ( 5 ). The initial dose of estradiol should not induce menstruation. In this occur, the estradiol dose should be increased gradually over a period of two years to reach the adult dose of 2-4 mg micronized PO daily (or 100-200 mcg transdermal daily) ( 5 ). Evaluate mood and behavior before starting therapy and during titration. Evaluation with estradiol, gonadotropins and inhibin B may be performed as an indicator of fertility ( 5 ).

Boys, commonly have cryptorchidism, hypoplastic scrotum, micropenis and are usually infertile ( 1 ). Stimulation with human chorionic gonadotrophin (hCG) to promote testicular descent may be considered in order to avoid surgical correction and general anesthesia ( 43 ). Administration of hCG may also increase testicular size and length of the penis, which may improve the results of orchidopexy and later facilitate standing urination ( 43 ). There is no published data on the efficacy of this practice in patients with PWS. Surgical correction of cryptorchidism should be completed on the first or at the latest in the second year of life ( 30 )

Treatment of hypogonadism is initiated after the age of 14 years with intramuscular testosterone 50 mg, IM, once a month; increasing by 25-50 mg, every 6 months, as needed; or applying it transdermally (patch or gel preparation) ( 5 ).

## OBESITY

Obesity is the most common cause of metabolic complications and worsening of quality of life in individuals with PWS ( 44 ). Hyperphagia and obesity arise after an initial phase of low weight and failure to grow ( 44 ).

The PWS patients’ weight begins to increase between 18-36 months of age, without being associated with a significant increase in food intake ( 44 ). The onset of obesity is observed before a substantial increase in food intake. Children with PWS require 20% to 30% lower energy intake than healthy children of the same age ( 44 ). Individuals with PWS are classified into 7 nutritional stages that differ clinically according to age range ( Table 2 ) ( 45 ).

**Table 2:** Prader-Willli syndrome: Clinical Characteristics of the Nutritional Phases Phases (3)

Age	Nutritional phase	Clinical findings
Fetal-newborns	0	Decreased fetal movements (85%), low birth weight.
0-9 months	1 a	Hypotonia with difficulty feeding, weak, uncoordinated suck, needs assistance with feeding, oral feeds are slow, severely decrease appetite, may develop failure to thrive.
9-25 months	1 b	No difficulty feeding, growing appropriately on growth curve, normal appetite, no longer needs assisted feeding.
2.1-4.5 years	2 a	Weight increasing without an increase in appetite or excessive calories, will become obese if given the recommended daily allowance, needs to be restricted to 60%-80% of the recommended daily allowance to prevent obesity.
4.5-8 years	2 b	Increased appetite, increased interest in food, can feel full, will eat more than a typical child if allowed, will eat food in their line of sight if unattended, will become obese if allowed to eat what they want, will stop eating voluntarily.
8 years- adulthood	3	Hyperphagic, rarely feels full, constantly thinking about food, while eating one meal they are already thinking about the next meal, will continue eating if portion size is not limited, will steal to pay for food, can eat food from garbage, will gain considerable amount of weight if not supervised, food needs to be locked up, temper tantrums related to food, needs 50%-60% of the recommended daily allowances to maintain weight.
Adulthood	4	Appetite may still be increased or may be normal, can feel full, improvement in the control of appetite, not as preoccupied with food.

Several mechanisms have been proposed to explain the etiology of obesity in PWS, which include discontinuation of the satiety control hypothalamic pathways, changes in hormones that regulate food intake, and reduced basal metabolic rate. The exact mechanism for the development of obesity is still unknown ( 44 ). Changes in the satiety center; alteration in the hormonal circuits, food intake increased and hormonal deficiencies can contribute to affect the energy balance ( 46 ). Other factors implicated in the development of obesity include impaired muscle tone and body composition with a higher percentage of fat mass ( 47 ).

Functional resonance data suggest that patients with PWS present greater stimulation in post-meal sub-cortical (hypothalamus, amygdala and hippocampus) and in the food activation center in the limbic and paralimbic region compared to lean and obese controls without PWS ( 48 ). Obese people without PWS have a significantly higher activity in the dorsolateral pre-frontal and frontal cortex that is associated with better inhibition control of food intake ( 49 ). This response is even more intense for high-calorie foods ( 50 ). This indicates that the dysfunction of reward circuit regions associated with changes in satiety-regulating hypothalamic hormones is also involved in the process of weight gain ( 44 ). Following the hormones are altered in individuals with PWS and contribute to obesity:

- Ghrelin: it is synthesized mainly in the gastric fund and stimulates the hunger. The number of cells expressing ghrelin and the amount of ghrelin in the gastric background of people with PWS was 2 to 3 times higher than in the control group ( 51 ). Ghrelin is elevated in children with PWS, precedes the onset of obesity and is involved in the onset of the hyperphagic stage ( 33 , 52 ). In addition, ghrelin levels remain high and do not adequately suppress after food intake when compared to obese controls ( 53 );
- Insulin and PYY: are reduced and result in loss of stimulatory signals to the POMC neurons and loss of inhibitory signals for NPY neurons in the arcuate nucleus that does not stimulate α-MSH and β-MSH to control satiety pathway MCR438 activation ( 53 );
- Leptin: the role of leptin is still under investigation as evidence suggests no difference in leptin concentration in PWS and obesity ( 53 );
- TRH-TSH: Changes in this axis result in reduced energy expenditure;
- GH: Deficiency leads to reduced muscle mass and increased body fat;
- Obestatin: produced in the stomach by the post-translational modification of ghrelin. In contrast to ghrelin, obestatin suppresses food intake, inhibits gastric emptying, and decreases weight gain. Unlike ghrelin, obestatin binds to a G protein-coupled receptor, although it does not cross the blood-brain barrier ( 54 ). A significant difference was observed in plasma obestatin levels between patients with PWS and non-PWS obese;
- Pancreatic polypeptide (PP) and peptide YY (PYY) are reduced in children with PWS and have been shown to decline with age ( 54 ). Presently, the only control available for hyperphagia for most people with PWS is in external control by tutors and/or health care providers who take care of the child. Access to food should be restricted to ensure reduced caloric intake.

## METABOLIC SYNDROME

The development of metabolic syndrome and complications of obesity in children and adults with PWS are largely dependent on the degree of obesity ( 55 ). The frequency of insulin resistance, hyperlipidemia and hypertension in children with PWS is common but these conditions are more rare than in obese children without PWS ( 56 ). Some studies have shown that obese children with PWS have greater insulin sensitivity and less fatty liver disease compared to obese controls ( 57 , 58 ). These results have also been reported in adulthood ( 59 ). This is probably due to increased subcutaneous fat and decreased visceral fat deposition as well as the increase in adiponectin, which works as a protective factor in the development of metabolic syndrome ( 58 ).

Presently, the only control available for hyperphagia for most people with PWS is in external control by tutors and/or health care providers who take care of the child. Access to food should be restricted to ensure a reduced caloric intake.

## EARLY ADRENARCHE AND PRECOCIOUS PUBERTY

The levels of androgens in PWS may be slightly elevated during childhood, but these normalize in adulthood ( 60 , 61 ). Early adrenarche occurs in 30%-40% of children with PWS ( 58 ). Premature adrenarche in PWS is not rapidly progressive or associated with other signs of central puberty, and usually develops benignly ( 60 ). Investigations or additional treatments are not normally required. True precocious puberty is very rare in patients with PWS ( 60 ).

## HYPOTHYROIDISM

In PWS, hypothalamic dysfunction may lead to central hypothyroidism. There are several reports on the prevalence of hypothyroidism in SPW; with rates varying from 2%-30% ( 21 , 46 , 62 ). Data on thyroid hormone levels in children with PWS undergoing rhGH therapy are very limited. Doses of free T4, T3 and TSH should be monitored regularly ( 63 ). Festen and cols. evaluated 79 children with PWS showing a decrease in free T4 after the onset of rhGH (although still within the normal range). However, TSH remained normal and total T3 levels were normal. This may be due to an increased conversion of T4 to T3, which may be caused by rhGH therapy ( 64 ). Treatment with levothyroxine at usual doses for age and weight should be initiated if there is evidence of hypothyroidism.

Assessment of thyroid function is advisable prior to initiation of rhGH therapy as well as periodically during treatment ^5^ Biotin nutritional supplementation may interfere with the test, therefore, individuals should be without this supplement for one week prior to collection ( 5 ). A TSH level > 10 mUI/L combined with a free T4 level below the lower limit of the reference range indicates primary hypothyroidism. TSH below the lower limit of the reference or within the reference range combined with a low T4 level may indicate central hypothyroidism. Abnormal borderline results should be repeated for confirmation ( 5 ).

## ADRENAL INSUFFICIENCY

Children and adults with PWS have increased risk of adrenal insufficiency (AI) due to generalized hypothalamic dysfunction probably due to inappropriate secretion of corticotrophin releasing hormone by the hypothalamus ( 65 ). Adrenal insufficiency may occur in individuals with PWS, but its incidence is still uncertain ( 65 ). There is a higher occurrence of AI in obese female patients with the genetic deletion subtype ( 30 ).

There is no consensus on the appropriate assessment and management of central adrenal insufficiency in PWS ( 66 , 67 ). During an acute illness or other stress situation may be useful dosing of serum cortisol and ACTH levels to confirm diagnostic. Most studies agree that some individuals with PWS have central adrenal insufficiency and therefore physicians should not hesitate to treat with hydrocortisone if there is a clinical suspicion of possible adrenal insufficiency, even only at critical times such as disease or surgery ( 68 ). However, an adrenal reserve assessment can be made by measuring cortisol and ACTH at 8 am; serum cortisol level ≥ 18 mcg/dL (500 nmol/L) is expected ( 5 ).

The most appropriate course in this case is to discuss with the family the possibility of adrenal insufficiency during stress and to provide patients with the corticosteroid card for a dose of hydrocortisone during an infectious process ( 65 ). GH inhibits 11β-hydroxysteroid dehydrogenase type 1 (11βHSD-1), resulting in a lower conversion of cortisone to active cortisol. Patients with PWS who using rhGH should be monitored for the possibility of precipitating an episode of adrenal insufficiency ( 65 ).

## TYPE 2 DIABETES MELLITUS

Type 2 diabetes mellitus (T2DM) is common in adults with PWS reaching up to 25% of patients and rarely develops during childhood. Recommendations for screening for T2DM and metabolic syndrome are similar to general guidelines in obese adolescentes ( 30 ). Patients with PWS should have hemoglobin A1C, lipid profile, and transaminases investigated at baseline, then annually.

Treatment for T2DM or insulin resistance in PWS should also follow routine guidelines for the general population ( 30 ). Intensive diet counseling and regular exercise are the first-line approach. Metformin may be added as adjuvant therapy ( 69 ). The addition of other oral hypoglycemic agents and insulin may also be required based on hemoglobin A1c ( 70 ). More recently, treatment with GLP-1 agonists ( *e.g.* , exenatide, liraglutide) has been shown to have beneficial effects on weight, satiety, and glycemic control in adult PWS with T2DM ( 71 ). These medications have been well tolerated, possibly due to the higher threshold for pain and nausea in SPW ( 72 , 73 ). However, caution should be exercised because of the potential side effect of delayed gastric emptying and the risk of gastric rupture ( 70 , 72 , 74 ).

## DECREASED BONE MINERAL DENSITY

Historically, an increase in the frequency of osteoporosis and fractures in individuals with PWS has been reported. Fracture rates were reportedly to 29% in adults with PWS ( 75 ). Many studies examining bone mineral density (BMD) in adults with PWS found lower total BMD and high rates of osteoporosis ( 76 ). However, recent studies have not shown differences in BMD when compared to controls ( 77 ). Early onset of rhGH treatment has been associated with better BMD in adolescence ( 77 ). Probably, the decrease in bone mineral density during adolescence and osteoporosis in adulthood in PWS is due to hypogonadism ( 30 ).

In children and adolescents, BMD measurement should be evaluated in two sites – lumbar spine (L1-L4) and whole body, with the results expressed in Z-scores (mean values compared to age-matched controls). After 11 years of age in girls, and 14 years in boys, BMD should be evaluated as it correlates with low levels of sex hormones. Vitamin D and calcium replacement may be necessary ( 78 ).

## TRANSITION PERIOD FROM PEDIATRIC CARE TO CLINICAL CARE

During the PWS adult life, there is an increase in endocrinological problems; behavioral disorders and comorbidities associated with obesity. The probability of mortality at 29 years is 50% and at 42 years is 75% ( 79 ). Fortunately, current estimates of survival for individuals with PWS show that they are surviving further into adulthood. Therefore, health care in the transitional phase is important to maintain continuity of care and optimize treatment through the exchange of information among health professionals.

A recent study compared the endocrinological treatment and the anthropometric and metabolic parameters of adults with PWS, who had specialized pediatric follow-up and who did or did not have the scheduled transition. Patients in the transition group had improved metabolic and psychological parameters ( 80 ).

During the transition process the presence of obesity, diabetes, hypertension, heart disease, osteoporosis, sleep apnea, difficult behavior and psychiatric problems should always be considered ( 30 ). Weight reduction during the transition phase, even though difficult to achieve, remains one of the key issues in the prevention of morbidity and mortality ( 80 ).

The systematic endocrinological evaluation and neuropsychiatric evaluation at the end of the growth, still in the pediatric unit, before referring the patients to an adult department is the most indicated ( 80 ). Hokken-Koelega and cols. also recommend that a stimulus test be performed to evaluate GH secretion before the transition at the end of growth (between 14 and 15 years) ( 81 ).

In adult care, new healthcare settings should be centered on patient autonomy; changes in sexual development; psychological development; social problems; possibilities of work and financial aspects ( 80 ). It is hoped that children with PWS and their families will have the opportunity to meet with experts who can discuss and make suggestions about the transition into adulthood so that they continue to have lower prevalence of morbid obesity and mortality.

## FUTURE PERSPECTIVES: PHARMACOGENOMICS

The use of pharmacogenomic testing is becoming more common in clinical practice to check the status of cytochrome p450 required for normal drug metabolism. Evidence of changes in the genes involved in drug metabolism may affect drug selection as well as dosage ( 82 , 83 ). Some medications may be helpful in controlling obesity-related problems, but so far there are no successful drugs that control mandatory food intake. Depending on pharmacogenomics, weight gain is observed in individuals, with or without SPW, who use certain atypical antipsychotics as a risperidone.

### Oxytocin/cabernocin

Patients with PWS have fewer neurons in the paraventricular nucleus of the hypothalamus, and studies have demonstrated that plasma levels of oxytocin are decreased in patients with PWS ( 84 ). Interrupted signaling and feedback of oxytocin are hypothesized to play a role in symptoms of hyperphagia, anxiety, autistic characteristics, and obsessive-compulsive visualizations. In childhood, oxytocin indicates breastfeeding and help to improve diet; while later, oxytocin is a potent anorectic hormone related to homeostatic control of food intake, satiety and energy balance. Preclinical studies showed that oxytocin increases lipolysis and fat oxidation using visceral fat, liver fat and triglycerides ( 85 ). A single dose of intranasal oxytocin causes increased fat oxidation ( 86 ). Oxytocin also increases glucose uptake in skeletal muscles by activating an AMP-activated protein kinase (AMPK), This may explain how oxytocin administration helps preserve lean muscle mass and maintain lower energy expenditure from weight loss ( 87 , 88 ).

Further studies are needed to determine if the effects of oxytocin on the hypothalamic-pituitary-adrenal axis lead to a better metabolic profile in humans ( 5 , 39 ).

### Diazoxide

Diazoxide is used to treat hyperinsulinemic hypoglycemia. A long-acting preparation (diazoxide choline) is under investigation to treat hyperphagia by improving leptin and insulin sensitivity, especially in Agouti (AgRP) and pro-opiomelanocortin (POMC) neurons Clinical studies showed reduced hyperphagia, reduced fat mass and weight, improved resting energy expenditure, and improved antisocial behaviors ( 5 ).

### Glucagon like peptide 1 receptor agonists

Glucagon like peptide 1 receptor agonists (GLP- 1 ) and receptor agonists (GLP1RA), such as exenatide and liraglutide, are used for the treatment of type 2 diabetes. GLP1RAs promote weight loss and appetite suppression ( 89 ). Agonists when used in patients with PWS demonstrated decreased appetite scores, with antidiabetic effects, reduced body mass index, improved in satiety and decreased serum ghrelin ( 73 ).

### Melanocortin-4 receptor agonists

Heterozygous and homozygous mutations of the melanocortin-4 receptor (MC4R) cause monogenic obesity. MC4R receptor agonist RM-493, increases resting energy expenditure in obese adults ( 90 ). Recent trials in obese patients with heterozygous mutations in MC4R and other mutations via melanocortin hypothalamic signaling, as well as in individuals with PWS, look promising. A phase II trial of this drug in PWS was completed, but the data found no benefit ( 5 ).

### Unacetylated ghrelin analogs

Unacetylated ghrelin analogs inhibit acylated ghrelin plasma levels and improve glucose metabolism. Results showed decreased appetite, less food-seeking behavior and lower waist circumference ( 91 ).

In conclusion, PWS is a complex disorder involving the hypothalamic-pituitary axis, resulting in multiple endocrinopathies, among other problems. Monitoring and management of these conditions in children, adolescents and adults with PWS should be guided by endocrinologists. However, early diagnosis is an important issue for both endocrinologists and pediatricians and can change the course of the disease for those who have PWS.

In recent years, developments in scientific knowledge have improved clinical management with better results than survival. Although this document has focused on the endocrinological aspects of PWS, these patients require multidisciplinary follow-up and special attention should be given from the time of diagnosis to a well-balanced diet with caloric restriction, daily physical activity and early initiation of rhGH.

Several studies are investigating therapeutic alternatives for PWS, such as: oxytocin for better appetite control, weight and social skills; use of non-acetylated ghrelin analogs for the reduction of hyperphagia and the use of glucagon-like peptide analogs for the treatment of excess of weight and diabetes.
